# Sonication induced amorphisation in Ag nanowires

**DOI:** 10.1038/s41598-019-38863-6

**Published:** 2019-02-14

**Authors:** Han Dai, Haitao Li, Zhutie Li, Junfeng Zhao, Xinxiang Yu, Jie Sun, Qi An

**Affiliations:** 1 Laboratory of Advanced Light Alloy Materials and Devices, Yantai Nanshan University, Longkou, 265713 China; 2 Hang Xin Material Technology Co. Ltd, Longkou, 264006 China; 3 Beijing Key Laboratory of Materials Utilization of Nonmetallic Minerals and Solid Wastes, National Laboratory of Mineral Materials, School of Materials Science and Technology, China University of Geosciences, Beijing, 100083 China

## Abstract

It has long been conjectured that pure-element face-centred cubic (fcc) metals can be transformed into a glassy state by deformation at ultra-high strain rates. However, when an impact force is applied at the nanoscale, deformation-induced melting prevents observations of fcc metal amorphisation. Here we propose a sonication treatment of Ag nanowires (fcc) and confirmed amorphisation induced by high strain rates at bent areas of the Ag nanowires. Owing to the mismatch of the deformation modes between the core and the surface, we observed a diameter related increase of the ductility of Ag nanowires under deformation at ultra-high strain rates generated by sonication. The sonication-prepared amorphous Ag was stable at room temperature. Amorphous Ag at the bent areas was highly reactive and was readily recrystallized under light illumination or vulcanised. Our study verifies the occurrence of high strain rate induced amorphisation in pure fcc MGs and provides a powerful tool for mechanical studies on metal nanomaterials under extremely high strain rates and forces.

## Introduction

Since the discovery of metallic glass (MG) in the 1960s, a variety of alloyed MGs have been explored and found wide application in various fields^1–3^. Unfortunately, pure MGs are difficult to obtain because of their extremely low glass-forming ability and thermal stability at room temperature. Only amorphous Ni, Ge and Ta, V, and W and Mo have been obtained from their liquid states under extreme conditions (with the exception of alloys formed by atomic accumulation methods such as vapour, chemical deposition)^4–7^. For other metals, in particular face-centred cubic (fcc) metals (e.g., Ag, Au, Cu, Pd, and Al)^4^, nucleation of crystallisation is predicted to be spontaneous and fast, even at absolute zero, which increased difficulties in obtaining fcc pure MGs.

Goddard *et al*. theorised that single crystalline Ni and NiCu nanowires (fcc) can directly transform into MGs under extremely high strain rates (up to 5% ps^−1^) at constant temperature (300 K)^8^. Hemker *et al*. experimentally observed the formation of amorphisation in boron carbide under 907 m/s shock impaction, macroscopically^9^. However, direct comparisons with experiments are difficult to justify owing to the difficulty in conducting controlled laboratory experiments at high strain rates on nanowires to date^10–12^. The heat generated by the extremely high strain rates causes melting, which prevents observations of amorphisation.

Sonication is widely used in material synthesis to release entangled materials or induce impact between materials^13^. The effects of sonication are mainly induced by cavitation bubbles. An enormous concentration of energy is released from the conversion of the surface energy, which causes high-speed liquid motion (bubble jets), which flow at rates up to 1000 m/s and induce a pressure of several MPa to several tens of GPa on metal nanostructures^14,15^. Metals have ultra-short characteristic natural thermal diffusion times at the nanoscale, which can efficiently avoid heat accumulation in the metal nanostructures^16^. As a result, sonication exerted on metal nanostructures can induce ultra-high strain rates at the same time avoiding deformation-induced melting.

Excellent fcc metal nano-materials of varying diameters and lengths can be synthesized from Ag nanowires by a lot of methods, such as microwave, and hydrothermal synthesis^17^. Sonication assisted routes can also be applied for the synthesis of Ag nanowires^18–20^. It is worth noting that some Ag nanowires contain amorphous phases or bended nanostructures when obtained by the sonication assisted routes^19,20^. Regretfully, Ag nanowires by sonication have not been fully studied.

Herein, we propose the use of sonication of Ag nanowires in order to understand their deformation and properties under extremely high strain rates. Firstly, we experimentally confirmed amorphisation induced by the high strain rate at the bent areas in Ag nanowires by a sonication treatment. Secondly, we observed an exceptionally high ductility of the Ag nanowires during the sonication process. We found that the sonication-induced amorphisation of the Ag nanowires showed stability at room temperature. After a short period of illumination, we observed recrystallisation at the bent areas of the Ag nanowires. *In situ* chemical reactions also revealed a considerable increase of the chemical activity of the amorphous Ag on the Ag nanowire surface.

## Results Section

**Figure 1:**
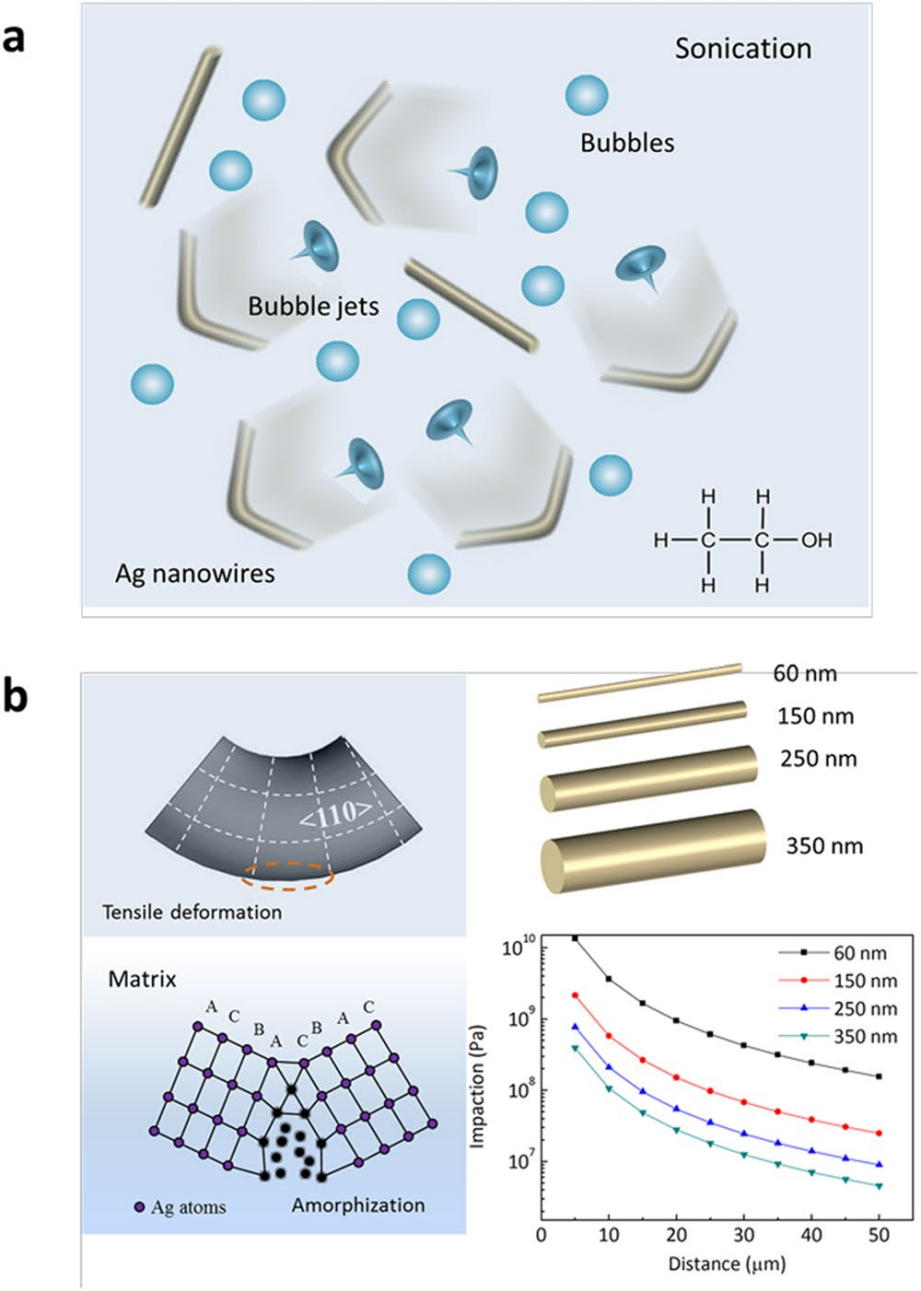
Illustration of Ag nanowire deformation under ultra-high strain rate induced by sonication. (**a**) Schematic showing how bubble jets induce pressure on Ag nanowires in ethanol. (**b**) Matrix transformation of the Ag nanowire surface; sonication effects on Ag nanowires with a length 40 μm and diameters of 60, 150, 250, and 350 nm.

Figure 1a shows the possible rupturing effects caused by the cavitation bubbles jets ejected towards the Ag nanowires in ethanol solution. The bubble jets exerted extreme forces on the nanowires. The ultrasonic strength exerted on the nanowires depended on the nanowire length, diameter, and the distances from the bubble jet. The flow rates of cavitation bubbles were directly proportional to the ultrasonic forces exerted on the nanowires. After a 2-min sonication treatment, bending deformation and breakage occurred at most of the Ag nanowires. For the bent Ag nanowires, the outer layers of the bent areas of the Ag nanowires were subjected to tensile stress and became elongated along the <110> crystal direction, due to the typical bending deformation of Ag nanowires, as shown in Fig. 1b. The outer layers of the bent areas showed the fastest deformation rates and maximum stress; hence, the amorphisation was observed particularly at the outer layers of the bent areas. The ultrasonic forces decreased rapidly as the distance from the bubbles increased, which indicated that the highest strain rate deformation of the nanowires should occur in close proximity to the bubbles. As shown in Fig. 1b, the impaction on nanowires with a length 40 μm, and a diameter of 60 nm reached up to 13 GPa. Even at a diameter of 350 nm, the pressure reached almost 0.4 GPa at a distance 5 μm away from the bubble jet. Detailed deduction can be found in our previous study^21^ and has been provided in the supporting information. Depending on the maximum flow rates surrounding the bubble^22^, the ultrasonic-induced strain rate of the nanowires reached up to 10^9^–10^11^ s^−1^. Therefore, sonication treatments of Ag nanowires provide an excellent platform for studying high strain rate deformation at the nanoscale.

**Figure 2:**
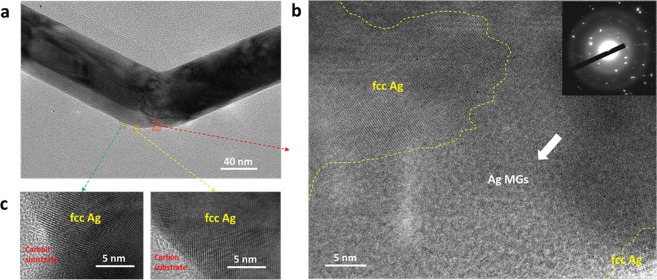
TEM results of the Ag nanowire matrix after ultra-high strain rate deformation. (**a**) Bent area of Ag nanowire. (**b**) Amorphous Ag on the nanowire surface. (**c**) Lattice distortion surrounding a bent area of the Ag nanowire surface.

As predicted by Goddard *et al*. the amorphisation of metal nanowires occurs at strain rates greater than 5 × 10^9^ s^−1^. The strain rates generated by sonication (10^9^–10^11^ s^−1^) should be enough to induce Ag amorphisation. We confirmed amorphous Ag at the bent areas of Ag nanowires prepared by a sonication treatment from TEM diffraction patterns, as shown in Fig. 2. The inset of Fig. 2b shows a small area, which includes Ag with a crystal phase from several electron-thin regions surrounding the bended area gave a similar diffraction pattern, thus confirming the formation of amorphous Ag. For the Ag nanowire of approximately 60 nm, the amorphous transformation layer of the Ag nanowire was several tens of nanometres in length and was often mixed with the crystal phase. As shown in Fig. 2b, we found two glass-crystal interfaces in the amorphous Ag, which indicated incomplete amorphisation or recrystallisation during the bending deformation. Serious lattice distortion was found away from the centre of the bent area and this distortion feature is a typical crystal deformation mode, as presented in Fig. 2c.

**Figure 3:**
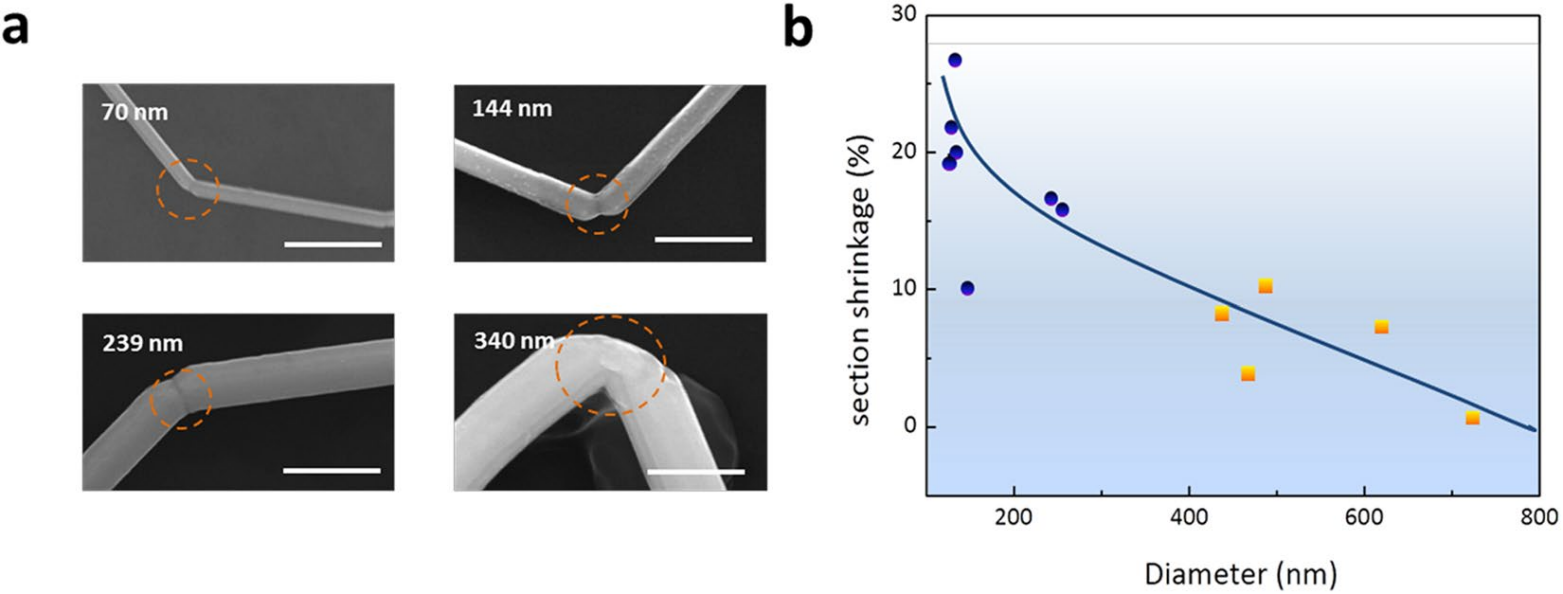
Ultrasonic bending deformation and section shrinkage at bent areas of Ag nanowires with various diameters. (**a**) Bending deformation of Ag nanowires with diameters of 70, 144, 239, and 340 nm; Scale bar is 500 nm. (**b**) Statistical analysis of section shrinkage at the bent areas of the Ag nanowires with diameters varies from 100 to 800 nm. Blue circles indicate nanowires without surface cracking and yellow squares indicate those with surface cracking.

The high strain rates induced amorphisation at the bent area, indicating that a stress of several tens of GPa acted on the region. However, abnormal bending deformation and section shrinkage rather than breakage occurred at the bent areas of the Ag nanowires, as shown in Fig. 3(a). The morphologies of the bent areas were clearly related to the diameters of Ag nanowires. As shown in Fig. 3(b), the relative shrinkage of the Ag nanowires with smaller diameters was more pronounced than in the case of those with larger diameters. Notably the deformation of Ag nanowires with diameters less than 250 nm was more homogeneous and occurred without catastrophic surface cracking. As the diameter increased to 400 nm, the bending deformation of the Ag nanowires was accompanied by serious surface cracking. These characteristics are marked in Fig. 3b, circled by blue points and squares, respectively.

## Discussion Section

Deformation-induced heating is a major barrier to obtaining MGs at ultra-high strain rates. However, careful calculation indicated that the heat generation will not accumulate in the defect, and the observed change was mainly induced by the ultra-high strain generated by sonication. Taking advantage of the ultra-short thermal diffusion distance of the metal at the nanoscale, the nature of thermal diffusion in Ag nanowires was limited to efficiently avoid heat accumulation. The characteristic time for thermal diffusion through an axial section of the Ag nanowire can be expressed as, $t={(d)}^{2}/16{\alpha }_{Ag}$, where *d* is the diameter of the Ag nanowire and *α*_*Ag*_ is the thermal diffusivity of Ag (1.76 × 10^−4^ m^2^ s^−1^)^16^. Assuming that the deformation heat generation occurs at the centre of the Ag nanowire (diameter 60 nm), the characteristic time is only 2.27 ps. The characteristic time of the thermal diffusivity is much greater than that of the strain rate, and this difference enables heat dissipation and avoids localised melting. Apparently, the enthalpy of fusion was overcome by the high strain rates induced by sonication rather than Boltzmann thermal energy. This effect led to the formation of amorphous Ag on the Ag nanowire surface. The strain rates decreased rapidly away from the centre of the bent areas of the Ag nanowires. As a result, the distortion feature away from the centre of the bent areas shows a typical crystal deformation mode.

The diameter related bending deformations and section shrinkages under extremely high stresses could be attributed to momentum-induced non-uniform amorphisation of the Ag nanowires, which resulted in an amorphous surface and a crystal core, as shown in Fig. 2(b). The distances of the accumulative dislocation release from the neutral axis to the top surface of Ag nanowires were much shorter in Ag nanowires with small diameters. In Fig. 2, for Ag nanowire with diameter 60 nm, the dislocations release distance is about 40 nm, which is quite short distance. The short distances enabled adequate release of the dislocations on the surface of the Ag nanowires to form amorphous surfaces before serious strain concentration and a great increase of their yield strain. This inadequate dislocation release generated micro-cracks in the Ag nanowires with larger diameters under high strain rate deformation. As previously reported, homogeneous deformation can exhibit plastic flow without work hardening or necking under deformation at a high strain rate at the surface^23^, which has considerably different deformation modes to those of the crystal core. Apparently, the mismatch in the regions of different deformation modes increased as the diameters of the Ag nanowire increased, leading to surface cracking of the Ag nanowires. As a result, Ag nanowires with smaller diameters exhibited higher ductility at high strengths and strain rates.

**Figure 4:**
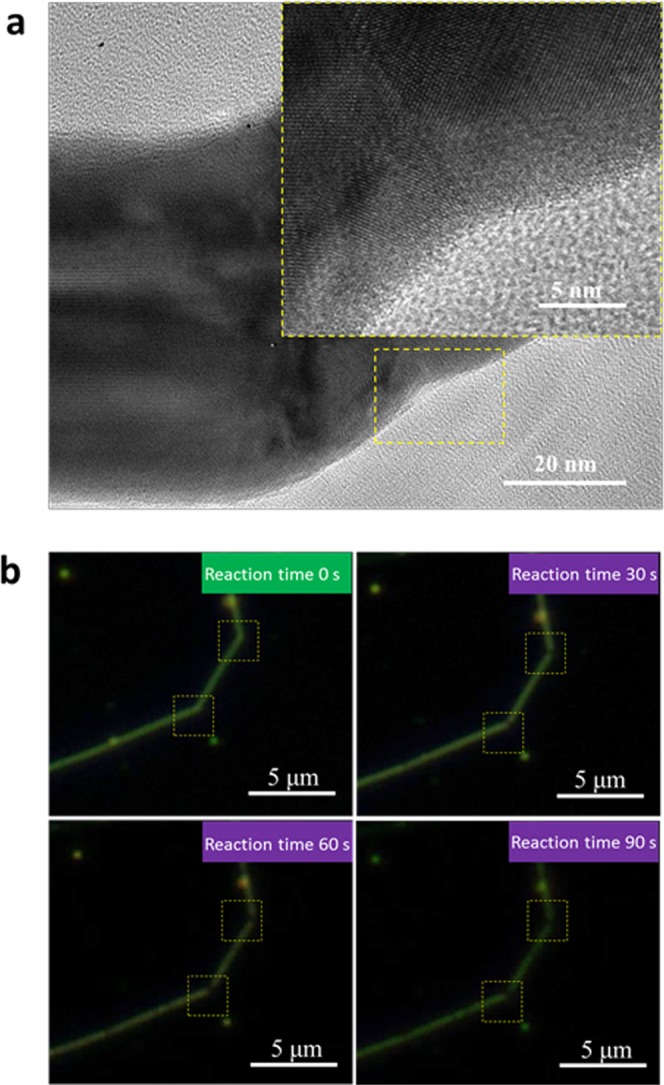
Stability of Ag nanowires in physical and chemical conditions. (**a**) Crystallisation of Ag nanowires under infrared illumination. (**b**) *In situ* vulcanization of Ag nanowire as indicated by regions enclosed by yellow boxes under an optical microscope.

The pure fcc MGs were thought to be unstable even at room temperature^4^, which led to the failed preparations by ultra-fast quenching methods. However, chemical methods have been used to prepare amorphous silver nanoparticles with diameters 20 nm by Gedanken *et al*., which exhibited crystallisation temperature up to 613 K^24^, which is far beyond the predicted temperature values. Our sonication-prepared amorphous Ag on Ag nanowires exhibited only room temperature stability. Total crystallisation occurred at the bent area of the Ag nanowire surfaces even after a short period of weak light illumination (infrared lamp 10 W/cm^2^ for 5 min), as shown in Fig. 4.

We also observed enhanced chemical activity surrounding the bent area. As shown in Fig. 4b, the black areas indicated by the yellow dot, covered the bent areas of Ag nanowire and grew bigger as the reaction was increased. Owing to the strong light absorption of silver sulfide in the visible spectrum, the vulcanisation (sulfur 0.01 wt%) rate of the Ag nanowires (black area growth) reflects the chemical activity of the Ag nanowires. As a result, Ag crystals with severe lattice defects surrounding the bent area exhibited higher chemical activity than that of the original Ag crystal. This phenomenon could provide a route to the design of metal catalysts at the nanoscale.

In summary, we report sonication induced amorphisation in Ag nanowires. The formation of amorphous Ag at the bent areas of Ag nanowires was confirmed from TEM diffraction patterns. Momentum-induced inhomogeneous amorphisation of the Ag nanowires led to diameter related bending deformation and section shrinkage of the Ag nanowires. The sonication-prepared amorphous Ag showed stability at room temperature. Furthermore, enhanced chemical activity was identified at the bent area and related to the formation of defects and electronic imbalance in the crystal lattice surrounding the bent area. This work opens a completely new approach to studying the mechanical properties of metal nanostructures under extreme deformation conditions. Considering the wide application of sonication in treatment of Ag nanowire-based flexible electronics, our work also provides insight in the changes these devices might experience during the sonication process.

## Methods Section

Ag nanowires with an average diameter of 60 nm and length of 40 µm were purchased from Nanjing XFNANO Materials Tech Co., Ltd. Through the SEM Mapping test, the properties of the commercial Ag nanowires can be well confirmed, as shown in Figs S1 and S2. HRTEM images of Ag nanowires on different positions have been presented after purification, as shown in Fig. S2. Others with a diameter ranging from 150 to 350 nm and length of 40 to 80 µm were synthesised by our hydrothermal method. PVP with an average molecular mass of 360 KDa, silver nitrate, and sodium sulfide were dissolved in ethylene glycol and then placed in a 50 mL Teflon-lined stainless autoclave. Ag nanowires of various sizes were obtained after 4–6 h under 160 °C. The purification of the Ag nanowires was performed by repeated ethanol centrifugation. All Ag nanowires exhibit good crystal quality. The Ag nanowires were suspended in ethanol and then sonicated at 1 s intervals for 2 min with 20 kHz, power 10 W/cm^2^ at 0 °C in ice bath. An ultrasonic probe (power 0–900 W) was used for in our experiment. A 25 ml beaker was adopted as reactor. The beaker was immersed in a larger beaker with about 50 ml ice water mixture to cool down their temperature. Ethanol is adopted because it is an easily available, cheap and lowly toxic dispersant of Ag nanowires, which reduces the effect of entanglement between nanowires during the sonication treatment. Moreover, the reducibility ethanol can effectively protect Ag nanowires from oxidation under the sonication conditions. In contrast to other organic solvents with high viscosity, ethanol with viscosity of 1.79 mPa · s (0 °C) should be a good choice to exert forces on nanowires.

The morphology and lattice of Ag nanowires were observed with an optical microscope (Model 10XB-PC; Yongxiang corp.), a field emission scanning electron microscope (SEM, Hitachi UHR FE-SEM SU8010) and a transmission electron microscope (TEM, JEOL JEM 2100 LaB6). Absorption spectra of the Ag nanowire dispersion solution were measured with a UV spectrophotometer (Hitachi S4800).

## Supplementary information

Sonication induced amorphisation in Ag nanowires
