# Supplementary material for: Sonication induced amorphisation in Ag nanowires
Source: Sci Rep. 2019 Feb 14;9:2114. doi: 10.1038/s41598-019-38863-6 (PMC6375950; doi:10.1038/s41598-019-38863-6)
Supplement: Supplementary file 1 — Sonication induced amorphisation in Ag nanowires [file 41598_2019_38863_MOESM1_ESM.pdf]

Supporting Information

Sonication induced amorphisation in Ag nanowires

Han Dai[1, 2*], Haitao Li[3], Zhutie Li[2], Junfeng Zhao[1], Xinxiang Yu[1, 2], Jie Sun[1], Qi An[3*]

[1]Laboratory of Advanced Light Alloy Materials and Devices, Yantai Nanshan University, Longkou, 265713, China.
[2]Hang Xin Material Technology Co. Ltd. Longkou, 264006, China.
[3]Beijing Key Laboratory of Materials Utilization of Nonmetallic Minerals and Solid Wastes, National Laboratory of Mineral Materials, School of Materials Science and Technology, China University of Geosciences, Beijing, 100083.

Contents

1. Deduction about the relation of bubble jets induces pressure on Ag nanowires;
2. SEM, SEM Mapping and TEM characterization of Ag nanowires.

1. Deduction about the relation of bubble jets induces pressure on Ag nanowires

"Based on our 'bubble-jet impacted' model, the vertical force F from bubble jet on the nanowire can be expressed as $F(S) = 0.5\rho a U(S)^2$. Where ρ represents the density of ethanol, S is the distance between the nanowire and the bubble wall. a represents the effective action area of Ag nanowires (defined as $a = d \cdot D_0$, where D_0 denotes the diameter of the bubble jet, about 2-3 μm, and is simplified to a constant here) and $U(S)$ represents the fluid velocity of the jet. In the free fluid field, $U(S)$ can be simply expressed by $U(S)/U_0 = 0.96/(0.29 + 2\beta S/D_0)$, where U_0 is the initial velocity of the bubble jet, here set to 110 m/s, β is an empirical parameter, often chosen 0.7-0.8. The stress σ on nanowire derived from F is thus: $\sigma = 8F(S)(L - D_0)/\pi d^3$.

2. SEM, SEM Mapping and TEM characterization of Ag nanowires

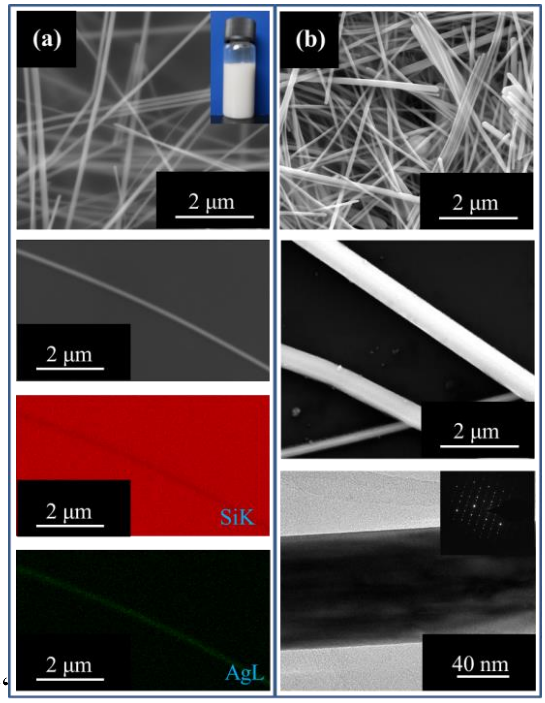

Figure. S1 The elements, morphologies of the purchased and synthesized Ag nanowires have been characterized by SEM Mapping and TEM. (a) Purchased Ag nanowires; (b) Synthesized Ag nanowires. Inset image in Fig. S1(a) is the Ag nanowire suspension in ethanol. Inset image in Fig. S1(b) is the image by selected area electron diffraction on Ag nanowire.

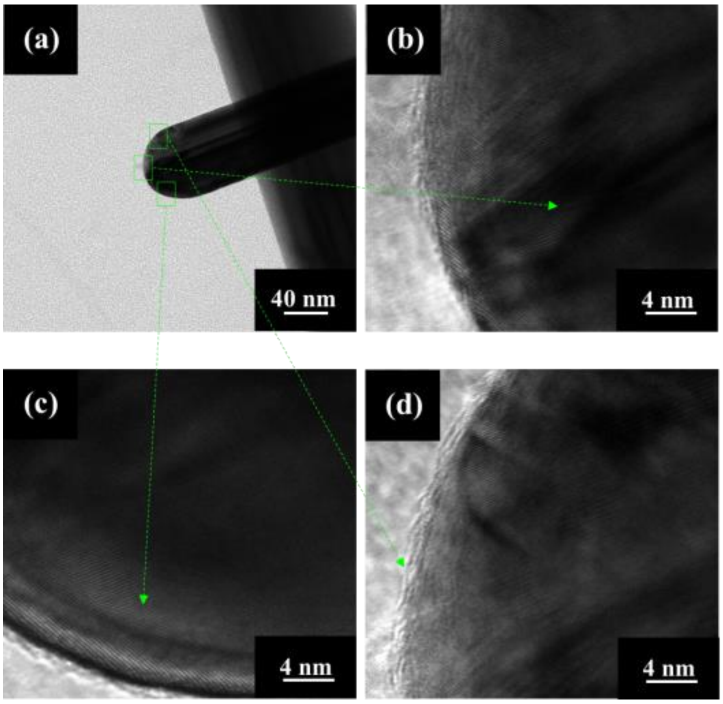

Figure. S2 HRTEM images of Ag nanowires on different positions after purification. (a) to (d) regular matrix on an end of Ag nanowire.
